# Oral Candidal carriage and associated risk indicators among adults in Sakaka, Saudi Arabia

**DOI:** 10.1186/s12903-019-0775-8

**Published:** 2019-05-22

**Authors:** Saad F. Alrayyes, Hammad M. Alruwaili, Ibrahim A. Taher, Khaled M. Elrahawy, Abdulrahman H. Almaeen, Awad O. Ashekhi, Mohammad Khursheed Alam

**Affiliations:** 1 Department of Pathology, College of medicine-Jouf University, Aljouf, KSA, Saudi Arabia; 2 College of Dentistry, Jouf University, Aljouf, KSA, Saudi Arabia; 3 College of Dentistry, Ajman University, Ajman, UAE

**Keywords:** *Candida spp*, Mouth rinses, Oral hygiene, Risk indicators

## Abstract

**Background:**

*Candida* is a ubiquitous organism in nature which inhabits the oral cavity as part of the normal microbial flora. The oral carriage of *Candida* is perpetuated by several predisposing factors.

**Methods:**

This cross-sectional study was designed to investigate the carriage rate of *Candida* among 104 voluntary adults at the college of medicine - Jouf University. The concentrated oral rinse technique using Sabouraud Dextrose agar medium supplemented with 0.05% Chloramphenicol was used to isolate *Candida*. The relative factors affecting the colonization of *Candida* and the concentration of each type were also determined.

**Results:**

*Candida* species were isolated from the oral cavity of 45 (43.4%) subjects. Of these 55.6% were identifies as *C. albicans* as determined by the Vitek 2 compact system. Other Candida species were represented by *C. glabrata* (11.1%), *C. krusei* (11.1%), *C. dubliniensis* (8.9%), *C. parapsilosis* (6.7%), *C. tropicalis* (4.4%), and *C. famata* (2.2%). Subjects with very poor plaque status, severe gingivitis and diabetes had significantly (*P* = 0.001) high concentration of *Candida* spp.

**Conclusion:**

Plague, severe gingivitis, and diabetes were found to be significantly associated with higher *Candida* colonization.

## Background

The term *Candida* originates from the Latin word “candid,” which means white. Following the emergence of the Human Immunodeficiency Virus (HIV) and the widespread usage of immunosuppressive therapy, there has been a wide interest among scientists in these white fungi [1, 2]. Of the over 200 known species of *Candida* only 40 are capable of causing disease. *Candida albicans* is the most prevalent isolated species in both symptomatic and asymptomatic candidiasis cases. In addition, *C. parapsilosis, C. krusei, C. tropicalis, C. glabrata, C. dubliniensis, C. lusitaniae,* and *C. kefyr*, which are collectively referred to as *non-albicans*, are increasingly involved in *Candida* cases and have been recognized as important pathogens [3, 4].

*Candida* are considered to be normal commensal organisms of the mouth that present most frequently in the posterior part of the dorsum of the tongue and can also be found on other body surfaces in the vagina and digestive tract [5, 6]. However, *Candida* are known to elicit disease only if there is debilitation to an individual’s immune status either locally or systemically [7]. In such cases, these commensals invade and penetrate the mucosal surfaces and form an essential step in the development of candidiasis [8]. Since candidiasis is often caused through endogenous infection by the *Candida* specie*s*, it is important to study and investigate the normal commensal flora of asymptomatic hosts at the population level.

The oral carriage rate of these organisms among the general population fluctuates between 3 and 75% without the appearance of any symptoms [9]. The disparity that has been noticed in the oral carriage rates of *Candida* might be influenced by multiple factors including demographic status, oral hygiene, and inconsistencies in the criteria of sample selection as well as in the analytical and sampling techniques used [6, 9, 10]. In addition, the carriage rates of oral *Candida* that have been reported to be more frequently associated with other factors are female gender, systemic diseases (e.g. diabetes mellitus), different blood groupings, tobacco smoking, and poor oral hygiene [11–14]. Additional factors such as geographical variations have also been suggested as affecting oral *Candida* carriage [9].

According to the information obtained from the indexed literature, data on asymptomatic oral *Candida* carriage in Saudi Arabia is limited to just one study [15] in which some aspects of oral *Candida* carriage and its relationship to poor oral hygiene were described in healthy Saudi subjects. Therefore, the objective of the present investigation was to study the carriage of *Candida* species (spp.) among adult individuals at Jouf University’s Medical College, in Saudi Arabia.

## Methods

This is a cross-sectional study that was carried out by performing clinical checkups, filling a questionnaire, and conducting a culture based microbiological investigation. Adult volunteers participated from both genders attending the college of medicine Jouf University. All candidates were given sufficient information about the study before being asked to formally consent to participate. The study protocol was also approved by the Medical Ethics Committee of the college of medicine, Jouf University. The population study was made of 104 adult individuals based on the calculation of a sample size measurement. The questionnaire contained questions on antibiotic/antifungal intake, and other health measurements and dental status.

## Collection of samples and identification of ***Candida*** isolates

The concentrated oral rinse technique as described by Samaranayake et al. [16] for oral Candidal sampling was used in the present study. In brief, each participant was requested to rinse his/her mouth thoroughly for 60 s with 10 ml sterile saline. The rinse was recollected into a sterile container, and was centrifuged for 15 min at 1700 g. The supernatant was discarded, and the pellet was dissolved into l ml sterile saline. After thorough mixing for 30 s, 100 mL of each sample was transferred onto two agar plates of Sabouraud Dextrose medium (SDA, Oxoid – Basingstoke, U.K) supplemented with 0.05 μg chloramphenicol and incubated for 48–72 h at 35 °C in air. All isolates that resembled *Candida* were gram stained and tested for Germ tube formation. Further identification of the yeast isolates was performed using the Vitek 2 compact 60 system (Biomerieux, France) according to the manufactures instructions.

## Oral assessment

A visual examination using a probe and dental mirror was employed to perform the dental health status of the participants by the same researcher throughout the study. The dental health status and DMFT index (decayed, missing, and filled teeth) were assessed as described by Schuller and Holst [17]. The plaque thickness at the gingival area, and the plaque index (PI) were measured using the Silness and Loe method [18]. While, the gingival index (GI), and the gingival condition were evaluated using the Loe and Silness method [19].

## Self-reported data

Information regarding age, gender, education or work status, oral health condition (history of dental caries), frequency of teeth brushing, and frequency of daily usage of mouth washes, smoking (current smoker, non-smoker) were all tabulated in a self-explanatory questionnaire. The body mass index (BMI, kg/m^2^), blood grouping (ABO, A, B, and O), presence of diabetes and hypertension were also recorded for all the participants.

## Statistical analysis

The results were statistically analyzed using the Statistical Package of Social Sciences (SPSS) version 22.0. The Student’s t-test or Mann–Whitney test (for non-normally distributed data) was used to compare the difference between the means; while the Chi-squared test was used to analyze the differences between frequencies in the different groups. The one-way ANOVA analysis was used to determine the correlation between the density of *Candida* and the plaque/gingival status. A value of *P* < 0.05 was considered significant.

## Results

### Clinical characteristics and demographic status

Oral rinses were obtained from 104 adult subjects, of which 79 were males (76%) and 25 were females (24%). As shown in Table 1, the mean age of the study population was (25 years, SD ± 10). Blood group O was the most common (44.2% among the study participants followed by groups A; AB and B). Only 12.5% of the study populations were smokers, 6.7% hypertensive and 5.8% were diabetics (Table 1).

**Table 1:** Characteristics of the study subjects and percentage (%) carriage rate of *Candida* spp.

	(Total number = 104) *n* (%)
Presence of Candida	
Yes	45 (43.4%)
No	59 (56.7%)
Candida spp.	
*C. albicans*	25 (55.6%)
*C. glabrata*	5 (11.1%)
*C. krusei*	5 (11.1%)
*C. dubliniensis*	4 (8.9%)
*C. parapsilosis*	3 (6.7%)
*C. tropicalis*	2 (4.4%)
*C. famata*	1 (2.2%)
Gender	
Male	79 (76%)
Female	25 (24%)
Age groups	
< 30	90 (86.5%)
≥ 30	14 (13.5%)
Obesity (BMI ≥ 25)	
Yes	48 (46.2%)
No	56 (53.8%)
Co-morbidities	
Diabetes mellitus	
Yes	6 (5.8%)
No	98 (94.2%)
Hypertension	
Yes	7 (6.7%)
No	97 (93.3%)
Smoking	
Yes	13 (12.5%)
No	91 (87.5%)
Blood grouping	
AB	17 (16.3%)
A	26 (25%)
B	15 (14.4%)
O	46 (44.2%)

### Carriage of *Candida species*

Out of the 104 subjects examined, 45 (43.4%) were found to be colonized with *Candida spp. C. albicans* was the most frequently encountered being isolated from 23 (21.9%) individuals and constituting (55.6.4%) of the total isolates (Table 1). The mean concentration count (density) of the *Candida* was 505 ± 1724 CFU/ml. Other Candida species were also isolated though less frequently as *C. glabrata* and C. *krusei* both being found in 5 (11%), 4 *C. dubliniensis* (8.9%), 3 *C. parapsilosis* (6.7), 2 *C. tropicalis* (4.4%), and 1 (2.2%) *C. famata*.

### Factors associated with colonization of *Candida* spp.

The oral Candidal carriage and concentration (density) among both males and females regardless of their ages (Table 3) was found to be not significantly different (*P* = 0.35). Other factors such as, BMI (obese vs. non-obese), hypertension, and smoking all had no significant effect on either carriage rate or concentration of *Candida* among the study group. Although, the prevalence of oral carriage rate of *Candida* was comparable between diabetic participants and non-diabetic participants [4/45 (8.9%) versus 41/45 (91.1%) respectively; *P* = 0.23], nevertheless, the mean carriage concentration of *Candida* was significantly higher among diabetic participants than their non-diabetic counterparts [4492 (SD ± 5629) versus 293 (SD ± 634) *P* ≤ 0.00; Table 3). Subjects with Blood group O were found to be associated with a higher frequency of oral *Candidal* carriage, while those with blood group A showed a higher amount (density) of *Candida* growth, however neither was significant (Table 2).

**Table 2:** Relationship between oral *Candida* colonization and oral hygiene status

	Carriage of *Candida* (*n* = 45)	*p*-value	Mean CFU (±SD)	*p*-value
Daily teeth brushing				
Irregular (intermittent)	12 (26.7%)	0.70	538 ± 202	0.93
Once	14 (31.1%)		770 ± 1786	
Twice	19 (42.3%)		798 ± 2905	
Daily usage of mouth wash				
Irregular (intermittent)	24 (53.3%)	0.46	882 ± 2630	0.82
Once	8 (17.8%)		720 ± 1008	
Twice	6 (13.3%)		71 ± 104	
More than twice	7 (15.6%)		377 ± 937	
History of dental caries				
Yes	28 (62.2%)	0.19	823 ± 2467	0.16
No	17 (37.8)		409 ± 763	
Plaque Index (PI)^a^				
Very good	10 (22.2%)	0.85	352 ± 111	0.01
Good	24 (53.3%)		563 ± 293	
Poor	8 (17.8%)		470 ± 166	
Very poor	3 (6.7%)		6480 ± 3741	
Gingival Index (GI)^b^				
No inflammation	15 (33.3%)	0.11	129 ± 240	0.01
Mild inflammation	23 (51.1%)		586 ± 1470	
Moderate inflammation	4 (8.9%)		499 ± 600	
Severe inflammation	3 (6.7%)		4197 ± 6480	
Decayed, Missing, and Filled Index (DMFT)^c^				
No Caries	5 (11.1%)	0.09	230 ± 337	0.40
D-component (Decayed teeth)	26 (57.8%)		539 ± 1386	
M-component (Missed teeth due to caries)	7 (15.6%)		1841 ± 4354	
F-component (Filled teeth due to caries)	7 (15.6%)		277 ± 430	

^a^ = PI: Very good < 0.1, Good = 0.1–0.9, Poor = 1.0–1.9, and Very poor = 2.0–3.0

^b^ = GI: no inflammation < 0.1, mild inflammation = 0.1–1.0, moderate inflammation = 1.1–2.0, and severe inflammation = 2.1–3

^c^ = DMFT: DT > 0, MT > 0 and FT > 0

Data represent mean CFU/ml of colonizing Candida, (±SD) and *P*-values

### Correlation between oral/dental hygiene and *Candidal* carriage

As shown in Table 2, the oral carriage rate and concentration of *Candida* spp. among those who brushed or used mouth wash on a regular basis was comparable, but not significantly different. Similarly, dental caries though was associated with a higher rate of *Candida* carriage; however, it was not statistically significantly different from those reporting no dental caries. Additionally, no significant differences in the oral *Candidal* carriage (*P* = 0.85) among the subjects with the different plaque status was revealed. There were also no significant differences regarding the prevalence of oral *Candida* between subjects with no gingival inflammation, mild, moderate or severe gingival inflammation status (*P* = 0.11). Nevertheless, the mean concentration of *Candida* concentration was increased significantly as a result of poor gingival status, and likewise due to a poor plaque status (*P* = 0.01, and *P* = 0.01 respectively) as revealed by ANOVA analysis.

## Discussion

Based on the very limited information regarding the oral carriage of *Candida* in our region [9] and to the knowledge obtained from the indexed literature, only one study has investigated the asymptomatic oral carriage of *Candida* [14]. Other studies have focused on *Candida* infections of the bloodstream, called Candidemia, or exclusively on diabetic groups or infants [19–21].

However, several studies [12–14] have shown wider variations of oral *Candida* carriage among healthy individuals. Also, Odds et al. [7] reported that the rate of *Candida* colonization found in the saliva or mouth washes of normal subjects ranges from 25 to 71.3%. In a healthy Saudi Arabian population, Darwazeh et al. [14] reported a 52% prevalence rate for total *Candida*, of which 80.8% was *C. albicans*. These rates are comparable to our present findings (43.3% total *Candida* and 55.6% *C. albicans*) and consistent with the prevalence rate of 53% reported by Patil et al. [22] among Indian adults. In addition, our results confirm that *C. albicans* is the most prevalent species in the oral cavity in comparison to other non*-Candida albicans*.

Some of the factors that were found to be associated with oral *Candida* colonization include age, gender, poor oral hygiene, smoking, and the presence of systemic diseases [2, 10–15, 22, 23]. Loster et al. [24] reported that oral *Candida* colonization was influenced by both age and gender; however, the subjects in this study were mainly denture-wearers.

A correlation between body mass indexes (BMI) and oral conditions has also been observed [25]. BMI was classified into the following three categories in the World Health Organization (WHO)‘s World Health Report 1995: overweight (BMI ≧ 25.1), acceptable weight (BMI > 18.5–25), and underweight (BMI ≦ 18.4). We attempted to analyze the relationship between BMI and oral *Candida* carriage, but find no correlation. It was observed that smoking can affect *Candida* colonization [26] by indirectly influencing the level of salivary glucose, which in turn leads to increased *Candida* growth and colonization [26]. Nonetheless, our study did not demonstrate any clear link regarding this phenomenon, nor did it affect the status of the mean number of *Candida* colonization. Our results were found to be consistent with previous studies [11, 12] as they found that tobacco smoking on its own influenced neither the carriage rate of oral *Candida* nor the number of colonizing *Candida*.

Although several studies that have been conducted focus on the association between A, B, and O blood groups and *Candida* carriage [13, 27], contradictory observations have been reported, and other results lacked significant findings [28]. Therefore, the relationship between *Candida* species and ABO blood grouping remains inconclusive. Bamford-Mason et al. [13] showed that there is a significant relationship between the blood group O and *Candida* colonization, and our results were consistent with this, as we found that individuals with blood group O had higher carriage rates than those with other blood groups. Although Shin et al. [29] showed that most oral *Candida* carriers had blood group A, but there was no significant differences between the different blood groups.

The role of *Candida* species as a precursor of disease among diabetic patients was first described by Odds et al. [23], who observed a higher rate of oral candidiasis in patients with diabetes mellitus (DM) than in healthy individuals [30, 31]. However, another study has contradicted this relationship between DM and oral colonization by *Candida* [32]. Our results showed a significantly (*P* = 0.00) higher *Candida* count (CFU/ml) among diabetics than non-diabetics, as shown in Table 3. Although, these findings are consistent with some previous investigations [31–33], other studies did not show any significant differences between DM and the number of colonizing *Candida* [34].

**Table 3:** Factors associated with *Candida* colonization

	*Candida* carriage (*n* = 45)	*P*-value	Mean CFU (±SD)	*P*-value
Gender				
Male	34 (75.5%)	0.93	759 ± 2247	0.35
Female	11 (24.5%)		382 ± 875	
Age				
< 30	38 (84.4%)	0.59	760 ± 2159	0.21
≥ 30	7 (15.6%)		157 ± 343	
Obesity (BMI ≥ 25)				
Yes	20 (44.4%)	0.82	441 ± 1416	0.37
No	25 (55.6%)		847 ± 2376	
Co-morbidities				
Diabetes mellitus				
Yes	4 (8.9%)	0.23	4492 ± 5629	0.00
No	41 (91.1%)		293 ± 634	
Hypertension				
Yes	5 (11.1%)	0.12	1609 ± 2660	0.20
No	40 (88.9%)		549 ± 1909	
Smoking				
Yes	6 (13.3%)	0.82	243 ± 324	0.20
No	39 (86.7%)		732 ± 2138	
Blood grouping				
AB	8 (17.8%)	0.71	276 ± 163	0.21
A	13 (28.9%)		1641 ± 3504	
B	7 (15.6%)		543 ± 1080	
O	17 (37.8%)		156 ± 297	

Data represent mean CFU/ml of colonizing Candida, (±SD) and *P*-values

Investigations into whether there is a relationship between hypertension and *Candida* colonization have also been conducted over the past 5 years [25]. Our study found that subjects who had mild hypertension had a prevalence rate of 71.4% for oral *Candida* carriage. There was also a higher mean number of colonies associated with the mild hypertensive subjects than their counterparts; however, neither of these findings was proven to be statistically significant. The relationship between hypertension and *Candida* colonization has yet to be clarified and might in fact be due to unhealthy lifestyles.

Several clinical investigations were performed to study the presence of a possible relationship between *Candida* colonization status and oral hygiene [12, 35]. In these studies, researchers observed an inversely relationship between better oral hygiene and reduced carriage rate of the number of colonizing cells of *Candida* spp. A recent study showed that a lower frequency of tooth-brushing was suggested to be associated with metabolic syndromes [36]. Other studies reported using mouth wash and aqueous extract of green tea led to reduction in the number of *Candida* cells [37]. On the contrary, our prospective study couldn’t demonstrate any association between frequencies of teeth brushing on a daily basis or the frequencies of usage of mouth wash on daily basis and *Candida* colonization.

In this study, we found no correlation between the plaque index status and the rate of *Candida* colonization. However, we found the concentration of *Candida* cells was significantly increased as a result of poor plaque index status (*P* = 0.001). We also couldn’t demonstrate any significant differences between oral Candidal carriage and gingival status. On the other hand, a significant increase in the density of the number of *Candida* colonies among subjects diagnosed with severe gingivitis (*P* = 0.001) was observed. These findings are in agreement with previous studies that reported a significant relationship between the presence of oral candidiasis and gingival erythema in patients diagnosed with HIV infection [38]. Other studies have reported no significant relationship between *Candida*, plaque and gingival status [12]. We didn’t found any significant correlation between caries history or poor DMFT scores and *Candida* colonization. Our findings regarding the increased density of *Candida* cells in subjects with very poor plaque status and/or severe gingivitis might substantiate other hypothesis which links *Candida* reluctance to preferably reside in subjects whose dental plaque is associated with poor oral hygiene, high level of plaque status, and presence of high level of gingival inflammation [38].

### Limitation of study

Using of self-reported data such as information on comorbidities (hypertension and diabetes) was based on recall basis.

## Conclusion

Of all the studied indicators none seem to affect the rate of oral carriage of *Candida* among our subjects. However, very poor plague status, severe gingival inflammation, and diabetes were significantly associated with higher density of colonizing *Candida spp.*

## Abbreviations

BMI: Body mass index
CFU/ml: Colony Forming Units per milliliter
DM: Diabetes mellitus
DMFT: Decayed, missing, and filled teeth index
GI: Gingival index
HIV: Human Immunodeficiency Virus
PI: Plaque index
SD: Standard Deviation
SDA: Sabouraud Dextrose Agar.
SPP: Species
